# Determination of Mechanical Properties of Epoxy Composite Materials Reinforced with Silicate Nanofillers Using Digital Image Correlation (DIC)

**DOI:** 10.3390/polym14061255

**Published:** 2022-03-21

**Authors:** Aleksandra Jelić, Milica Sekulić, Milan Travica, Jelena Gržetić, Vukašin Ugrinović, Aleksandar D. Marinković, Aleksandra Božić, Marina Stamenović, Slaviša Putić

**Affiliations:** 1 The Academy of Applied Technical Studies Belgrade, 11030 Belgrade, Serbia; abozic@politehnika.edu.rs (A.B.); mstamenovic@politehnika.edu.rs (M.S.); 2 Center of Excellence for Photoconversion, Vinca Institute of Nuclear Sciences, University of Belgrade, 11030 Belgrade, Serbia; milicasekulic88@gmail.com; 3 Innovation Center of Faculty of Mechanical Engineering, University of Belgrade, 11030 Belgrade, Serbia; milantravica93@gmail.com; 4 Military Technical Institute, Ratka Resanovica 1, 11030 Belgrade, Serbia; jrusmirovic@tmf.bg.ac.rs; 5 Innovation Center of Faculty of Technology and Metallurgy, University of Belgrade, 11030 Belgrade, Serbia; vugrinovic@tmf.bg.ac.rs; 6 Faculty of Technology and Metallurgy, University of Belgrade, 11030 Belgrade, Serbia; marinko@tmf.bg.ac.rs (A.D.M.); slavisa@tmf.bg.ac.rs (S.P.)

**Keywords:** epoxy resin, silicate nanofillers, tensile properties, 3D DIC, DMA

## Abstract

In this study, silicate nanofillers; dicalcium silicate, magnesium silicate, tricalcium silicate, and wollastonite; were synthesized using four different methods and incorporated into the epoxy resin to improve its mechanical properties. Characterization of the newly synthesized nanofillers was performed using Fourier-transformation infrared (FTIR) spectroscopy, X-ray diffraction (XRD), scanning electron microscopy (SEM), and transmission electron microscopy (TEM). The purpose of this study was to analyze newly developed composite materials reinforced with silicate nanoparticles utilizing tensile testing and a full-field non-contact 3D Digital Image Correlation (DIC) method. Analysis of deformation and displacement fields gives precise material behavior during testing. Testing results allowed a more reliable assessment of the structural integrity of epoxy composite materials reinforced using different silicate nanofillers. It was concluded that the addition of 3% of dicalcium silicate, magnesium silicate, tricalcium silicate, and wollastonite lead to the increasement of tensile strength up to 31.5%, 29.0%, 27.5%, and 23.5% in comparison with neat epoxy, respectively. In order to offer more trustworthy information about the viscoelastic behavior of neat epoxy and composites, a dynamic mechanical analysis (DMA) was also performed and rheological measurements of uncured epoxy matrix and epoxy suspensions were obtained.

## 1. Introduction

Epoxy resins are utilized in adhesives and coatings [1,2,3,4,5], composite materials [6,7,8,9,10,11], electronics [12,13,14], and other applications due to their unique characteristics, which include good adhesive qualities, dimensional stability, high chemical resistance, and mechanical properties [15].

Adding a small amount of fillers to epoxy resins is a method for improving their chemical, mechanical, and thermal characteristics in contemporary commercial applications. The issue, however, is to select suitable resin and filler that will operate synergistically to increase the new material’s characteristics over the underlying material [16]. Choosing the appropriate epoxy resin filler helps increase the new material’s mechanical characteristics (tensile strength, tensile modulus, impact resistance, etc.), flame retardancy, resistance to heat deformation, thermal conductivity, rheological properties, scratch resistance, etc. [17,18,19]. Due to their homogeneous dispersion, these particles, whether micro or nano, enhance the stiffness and toughness of polymer systems. For example, Sim et al. have prepared fly ash reinforced epoxy composite and investigated its properties. The results have shown improved mechanical properties due to lower particle size [20]. Chee and Jawaid have also investigated the influence of bifunctionalized MMT on the overall properties of epoxy composites (mechanical, thermal, dynamic mechanical properties). The investigations have shown that the addition of low concentrations of nanofillers (~1%) leads to a significant increase in the ultimate properties of the investigated materials [21]. Nevertheless, the strength and shape of the incorporated nanoparticles influence the mechanical properties of the prepared materials [20]. Better dispersion is related to optimal matrix-filler cohesion, which leads to an overall improvement in the mechanical strength of the polymeric nanocomposite material [22,23]. Ocher has been proposed by Bekeshev et al. as a low-cost reinforcement for epoxy resin-based composites. The authors discovered a rational filler content that improved the materials’ thermal stability, decreased their flammability, and raised their bending failure stress, tensile strength, and impact resistance. The improper interaction of the polymer matrix with filler particles was found to be the cause of the decrease in strength at a lower or higher optimal filler loading. The particles were packed more tightly at higher degrees of filling, and the composite became a uniform medium in which the fracture surface delayed interacting with individual particles [24].

Mineral fillers often enhance hardness, stiffness, dimensional stability, scratch resistance, and while decreasing impact resistance [25]. Poh et al. have used silica, mica, and calcium carbonate as mineral fillers for the epoxy resin to improve its mechanical and thermal properties. However, the research revealed improved thermal properties and the deterioration of tensile properties due to increased brittleness of the investigated materials [26]. Astruc et al. have effectively integrated two kaolin samples of varying particle size into a polymeric matrix and enhanced the microhardness values [27]. Tiimob et al. used eggshells and amorphous SiO_2_ (1:1 ratio) to synthesize β-CaSiO_3_ nanoparticles used as epoxy resin reinforcement. The results showed improvements in flexural strength, modulus, and toughness after incorporating 1–4 wt % of nanoparticles [28]. Karle et al. investigated the performance of epoxy composites containing Wollastonite (CaSiO_3_) micro-particles systematically inserted into an epoxy matrix for reinforcing purposes. It has been found that the addition of CaSiO_3_ particles to an epoxy resin with a micro-particle concentration of 1–2 wt % improved mechanical characteristics in comparison to neat epoxy resin [29]. The mechanical properties of wollastonite and carbon fiber reinforced epoxy composites were compared by Xian et al. The enhanced strength and toughness are thought to be due to the fine wollastonite particles’ small particle size, low aspect ratio, and high adherence to the epoxy matrix, while coarse wollastonite fillers and short carbon fibers degraded most mechanical parameters except the modulus [30]. The morphology of the epoxy matrix or the filler load impacted the deformation mode of epoxy-based composites [31]. Additionally, as compared to their micron-sized counterparts, the benefit of employing nanofillers is that a high degree of reinforcing may be obtained at low inorganic levels, resulting in stronger and lighter structures [19].

The purpose of this research is to investigate composite materials reinforced with silicate fillers: CaSiO_3_, Mg_2_SiO_4_, Ca_2_SiO_4_, and Ca_3_SiO_5_ synthesized using newly designed combustion methods. Tensile testing and a full-field non-contact 3D Digital Image Correlation (DIC) method. 3D Digital Image Correlation allowed the determination of non-contact 3D coordinates, displacements, and strains of the tested materials [32,33]. All tests were used to analyze the influence of incorporated particles and their content on the mechanical properties of novel composite materials. A dynamic mechanical analysis (DMA) was also conducted to provide further accurate information regarding the viscoelastic behavior of neat epoxy and composite materials.

## 2. Materials and Methods

As a starting material, fumed silica (99.8% purity), and nitrates: Ca(NO_3_)_2_·4H_2_O (99% purity), Mg(NO_3_)_2_·6H_2_O (98% purity), and urea (NH_2_CONH_2_, 98% purity) were obtained from Alfa Aesar (Ward Hill, MA, USA). Oxalyl dihydrazide (C_2_H_6_N_4_O_2_, ODH, 98% purity) was acquired from Sigma-Aldrich (Burlington, MA, USA). The citric acid (C_6_H_8_O_7_, CA, 99.5% purity), and ethylene glycol (C_2_H_6_O_2_, EG) were delivered from Fisher Scientific International Inc. (Hampton, NH, USA), and Honeywell (Charlotte, NC, USA), respectively. Nitric acid (HNO_3_, grade 65%) was obtained from Macron Fine Chemicals™ (Radnor, PA, USA). Colloidal solution of SiO_2_ in ethylene glycol was prepared using Alfa Aesar (Ward Hill, MA, USA), silicon (IV) oxide, 30% colloidal dispersion in ethylene glycol, 0.02-micron particles.

All epoxy resin components were provided by Epoksan, Čačak. Plain epoxy was based on the mixture of bisphenol-A (Araldite GY 250, Huntsman, Salt Lake City, UT, USA), aliphatic monofunctional epoxy reactive diluent resin Araldite DY-E (Huntsman, Salt Lake City, UT, USA), and a curing agent Aradur 2963-1 (Huntsman, Salt Lake City, UT, USA). Table 1 depicts the main properties of resin and hardener.

Ca_2_SiO_4_ (dicalcium silicate, Larnite, DS) and Mg_2_SiO_4_ (magnesium silicate, Forsterite, MS) were synthesized using a simple combustion solution approach with oxalyl dihydrazide as a fuel [37]. Ca(NO_3_)_2_·4H_2_O, Mg(NO_3_)_2_·6H_2_O, and fumed silica were stoichiometrically combined (Equations (1) and (2)) with ODH, and dissolved in a minimum amount of double-distilled water, then well stirred. A ceramic crucible containing this solution was placed for 30 min in a pre-heated muffle furnace at 800 °C. The gel subsequently turned into a white foam that expanded to fill the vessel, leaving a white powder with an extremely porous structure.

(1) $$2\mathrm{Mg}{\left({\mathrm{NO}}_{3}\right)}_{2}+{\mathrm{SiO}}_{2}+2C_{2}H_{6}N_{4}O_{2}\to {\mathrm{Mg}}_{2}{\mathrm{SiO}}_{4}+6N_{2}+6H_{2}O+4{\mathrm{CO}}_{2}$$

(2) $$2\mathrm{Ca}{\left({\mathrm{NO}}_{3}\right)}_{2}+{\mathrm{SiO}}_{2}+2C_{2}H_{6}N_{4}O_{2}\to {\mathrm{Ca}}_{2}{\mathrm{SiO}}_{4}+6N_{2}+6H_{2}O+4{\mathrm{CO}}_{2}$$

Ca_3_SiO_5_ (Tricalcium silicate, Alite, TS) was synthesized utilizing the solution combustion process using urea as the fuel [37]. As starting materials Ca(NO_3_)_2_·4H_2_O, fumed silica, urea (NH_2_CONH_2_), and HNO_3_ were used. Following the chemical reaction (Equation (3)), all of the ingredients were dissolved in double-distilled water (10 mL of water were used for 1 g of sample) and stirred at 80 °C to evaporate the surplus water until gel was formed. The gel was placed in a ceramic crucible and heated for 30 min at 600 °C. As is common for conventional combustion synthesis, fluffy white powder was obtained during the first few minutes of heating.

(3) $$3\mathrm{Ca}{\left({\mathrm{NO}}_{3}\right)}_{2}+{\mathrm{SiO}}_{2}+5{\left(\mathrm{NH}\right)}_{2}\mathrm{CO}+4{\mathrm{HNO}}_{3}\to {\mathrm{Ca}}_{3}{\mathrm{SiO}}_{5}+3{\mathrm{NO}}_{2}+5{\mathrm{CO}}_{2}+12H_{2}O$$

**Figure 1:**
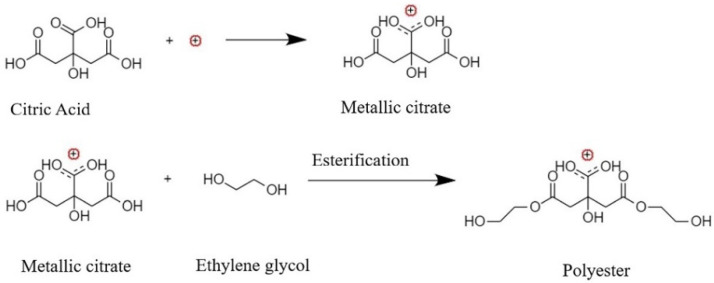
Reactions of wollastonite nanofillers synthesis by modified Peccini synthesis reaction [32].

CaSiO_3_ (Wollastonite, W) was synthesized utilizing a modified Peccini synthesis reaction (Figure 1), which was described elsewhere [38]. CA and EG were stirred in stoichiometric ratio to metal ions (Ca:CA:EG = 1:5:25) at 60 °C until completely dissolved. Ca(NO_3_)_2_·4H_2_O and colloidal solution of SiO_2_ in EG were added to this solution and stirred at 130 °C for several hours. A viscous yellow-transparent gel is created and heated for 2 h at 800 °C before being finally calcined at 1000 °C to form pure white powder [39].

Mechanical mixing of epoxy resin and various ratios of synthesized silicates was used to create the composite materials. Table 2 lists the fillers used, as well as the filler content and sample symbols.

**Figure 2:**
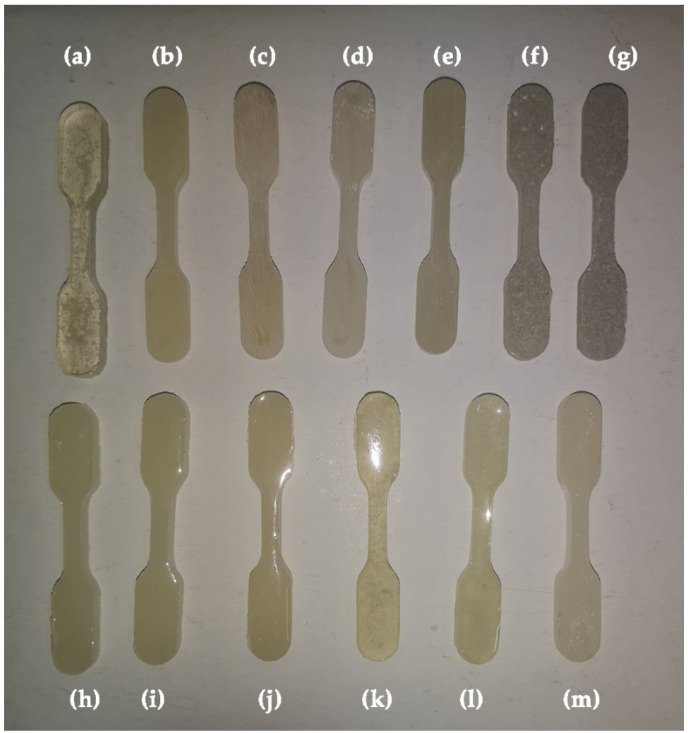
Plain epoxy and newly synthesized nanocomposites (**a**) PE, (**b**) PE/1DS, (**c**) PE/2DS, (**d**) PE/3DS, (**e**) PE/1MS, (**f**) PE/2MS, (**g**) PE/3MS, (**h**) PE/1TS, (**i**) PE/2TS, (**j**) PE/3TS, (**k**) PE/1W, (**l**) PE/2W, and (**m**) PE/3W.

To make homogenous mixtures, both amine and epoxy components (40:100) were blended at room temperature, and particles were added in various ratios. To avoid bubbles, the resulting mixture was kept at 35 °C for 30 min after mixing. The mixtures were put into a mold and degassed for 6 h. The samples were kept at room temperature for 12 h and then at 60 °C for 6 h until they were cured. Figure 2 depicts the created composites and plain epoxy.

A Nicolet iS 10 spectrometer (Thermo Fisher Scientific) was used in attenuated total reflectance (ATR) mode at 4 cm^−1^ resolution with ATR correction and OMNIC software for the Fourier transformation infrared spectroscopy (FTIR) analysis. The observed silicates’ spectra were recorded in the wavelength range of 4000 to 400 cm^−1^ using 32 scans.

To evaluate the crystallographic phase of the silicates, X-ray diffraction (XRD) assessment was conducted on the Rigaku SmartLab system using Cu Kα radiation supplied at 30 mA and 40 kV in the 2θ range from 10° to 90°at a scanning rate of 2° min^−1^ with 0.02° steps.

**Figure 3:**
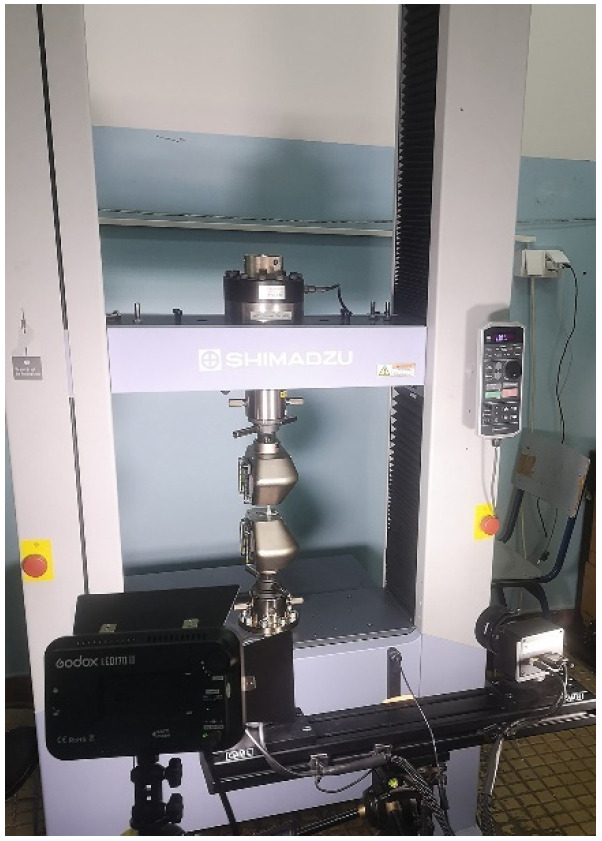
Experiment setup for tensile testing using 3D DIC.

The mechanical characteristics of the prepared specimen were investigated using the Aramis 2M 3D optical system (GOM, Braunschweig, Germany) and the Shimadzu Autograph AGS-X Series tensile testing machine (Shimadzu, Kyoto, Japan) with a maximum load of 1 kN at ambient temperature (approximately 23 °C) and a constant cross-head speed of 1 mm/min. The system consisted of two optical cameras offering synchronized stereo images of the specimen, a sensor stabilization stand, a power management system, an image storage unit, and data processing unit. Before recording, a layer of white paint (Kenda Color Acril 207ico, Kenda Farben, Garlasco, Italy) was applied to the recorded surfaces of the materials. The tests were carried out the following ASTM D 882, the American Society for Testing and Materials’ standard. Figure 3 shows the experiment setup for tensile testing and 3D Image Correlation. Seven samples from each group of materials were tested. The tested materials’ dimensions were according to the previously mentioned ASTM D 882 standard: 80.0 mm × 10.0 mm × 4.0 mm (the test length was approximately 38.0 mm).

In order to conduct a comprehensive analysis of the morphology of epoxy composite materials reinforced with silicate fillers, a transmission electron microscope (TEM) JEM-1400 with an accelerating voltage of 120 kV was utilized. Software Image-Pro Plus 6.0 was used to obtain the statistical data of the grain dimension.

Dynamic mechanical analysis (DMA) study of the plane epoxy and cured composite samples was performed in torsion deformation mode using the Modular Compact Rheometer MCR–302 (Anton Paar GmbH, Graz, Austria) equipped with standard fixtures (SRF12) for rectangular bars, temperature chamber (CTD–620) having high-temperature stability (±0.1). The standard sample of a rectangular bar shape (44 mm × 10 mm × 4 mm) was tested by using ‘‘temperature ramp test’’ at a temperature range from 40 °C to 180 °C, the heating rate was 5 °C·min^−1^, and the single angular frequency of 1 Hz and strain amplitude was 0.1%.

Rheological measurements of uncured epoxy matrix and epoxy suspensions with 3.0% of nanofiller content were carried out at 25 °C using a Modular Compact Rheometer MCR–302 (Anton Paar GmbH) equipped with standard 50 mm diameter parallel plates. A separation of 0.3 mm between the plates was used for all the experiments.

## 3. Results

### 3.1. Characterization of Silicates

**Figure 4:**
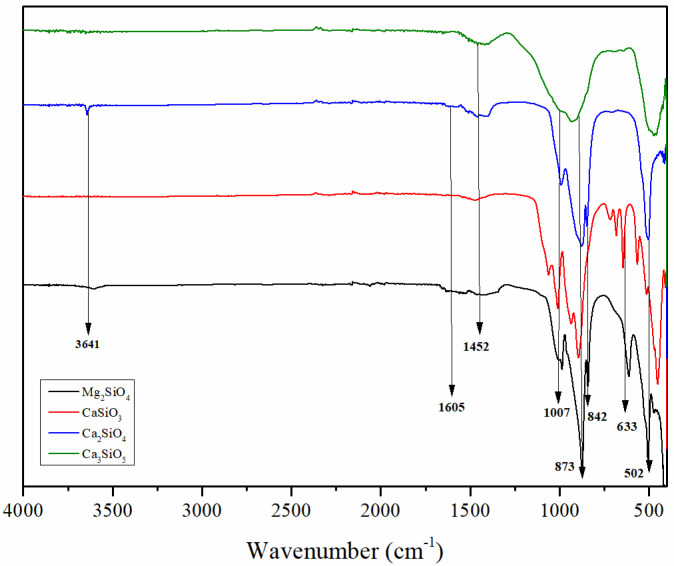
FTIR spectra of the prepared silicate fillers.

The structure of the synthesized silicates, CaSiO_3_, Mg_2_SiO_4_, Ca_2_SiO_4_, and Ca_3_SiO_5_, was confirmed in Figure 4. Asymmetric stretching of Si-O-Si bonds and their vibrations were suggested by peaks at 1007, 1055, and 982 cm^−1^ [40]. The occurrence of peaks at 982, 873, 832, and 611 cm^−1^ indicates symmetric and asymmetric stretching vibrations of SiO_4_ [41]. The vibrations of Si-O bonds in tetrahedra silicates at 676 and 642 cm^−1^ support the crystalline structure of silicates. C-O bonds specific for calcium silicates are demonstrated by the peaks at 873 and 707 cm^−1^. Mg-O vibration octahedra cause a strong peak at 502 cm^−1^ [42,43,44]. Furthermore, the vibrational characteristic of the hydroxyl groups is represented by bands at 1600 to 1450 cm^−1^, and 3641 cm^−1^ [45]. Those hydroxyl groups reflect the degree of functionality of the particles/fillers, and they are responsible for establishing hydrogen bonding interactions with the cured epoxy matrix, thus contributing to the reinforcing of nanocomposites.

**Figure 5:**
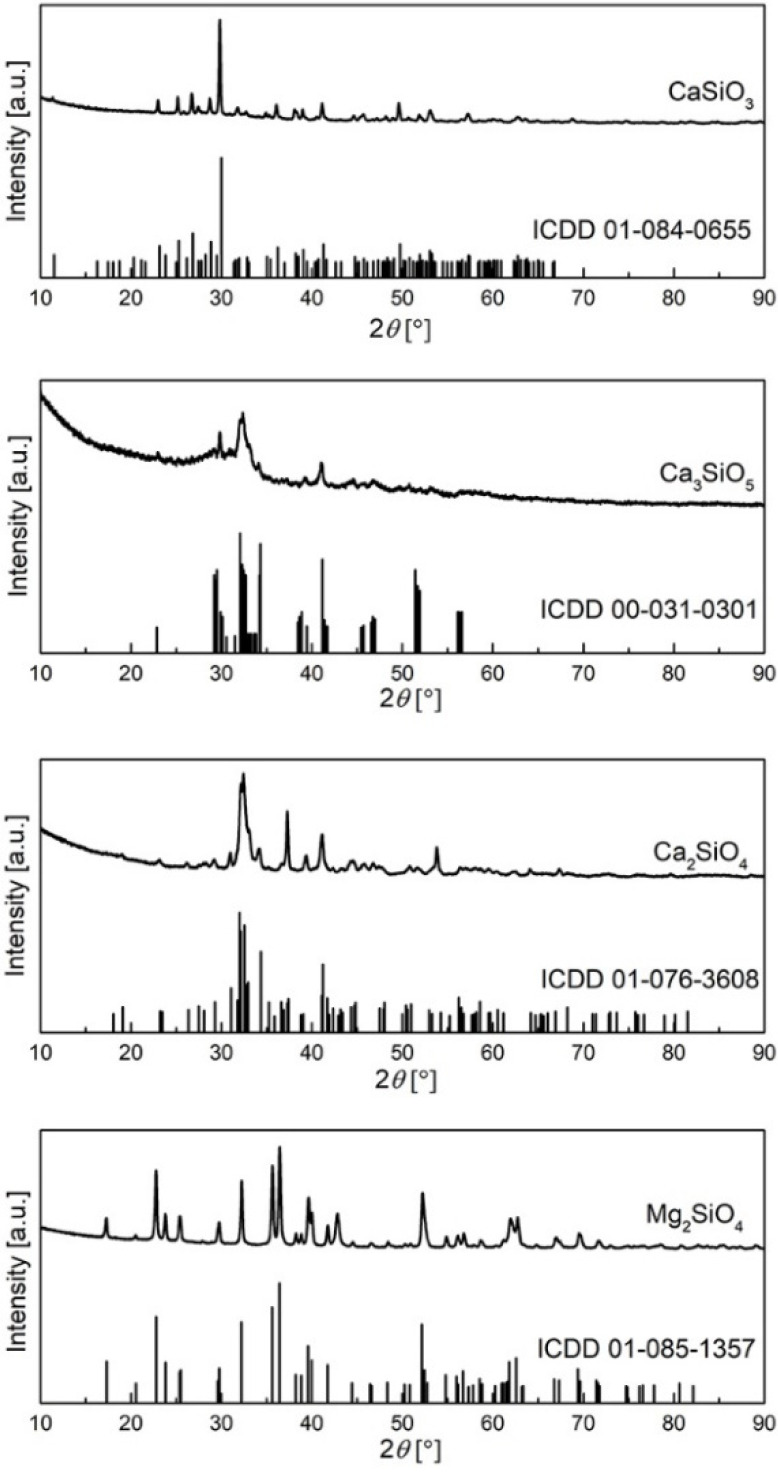
XRD spectra of the prepared silicates nanofiller.

The diffraction patterns of the samples are represented in Figure 5, and as can be observed, all peaks are matched to the appropriate card, with no additional peaks evident, suggesting that no other phase is present. The Rietveld refinement approach was used to determine mean crystallite size and structural parameters, and the results are reported in Table 3. The average crystallite size of the samples is estimated to be about 20 nm.

**Figure 6:**
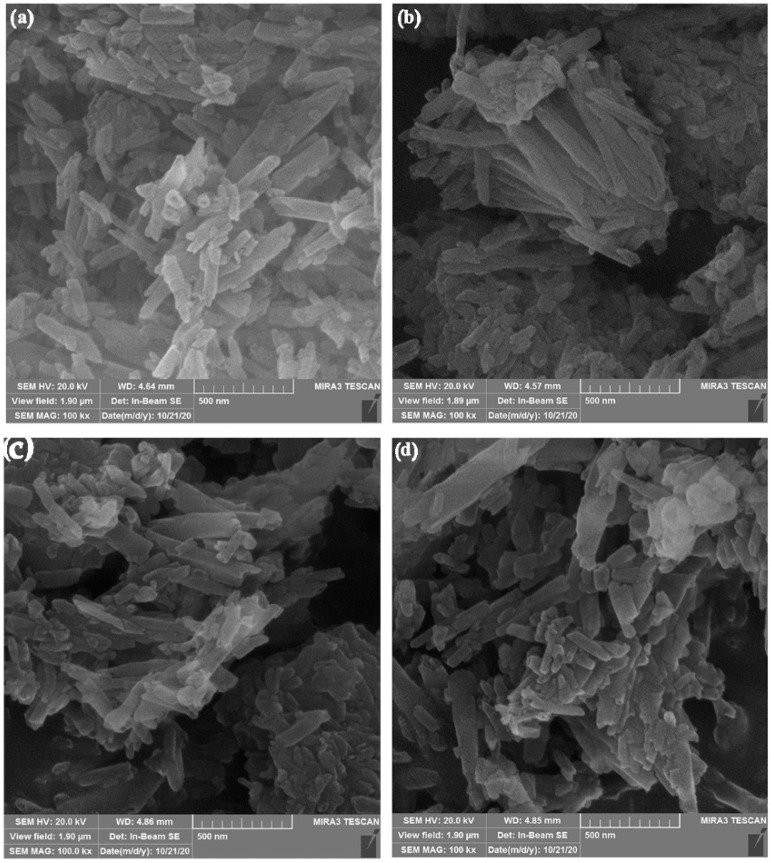
SEM images of silicates: (**a**) dicalcium silicate, Ca_2_SiO_4_, (**b**) magnesium silicate, Mg_2_SiO_4_, (**c**) tricalcium silicate, Ca_3_SiO_5_, and (**d**) wollastonite, CaSiO_3_.

Images of the silicates were taken at 100 kx magnification during SEM analysis. The primary goal of scanning photographs at this magnification level was to investigate the adherence of particles in the micrometer/nanometer range in the samples. The results of SEM analysis showed needle-like structures of the obtained silicates (Figure 6) with different nanometer and submicron dimensions. The results obtained by the Rietveld refinement approach are presented in Table 3.

Image ProPlus6.0 software was used to examine the silicate’s grain dimensions. Synthesized Ca_2_SiO_4_ (DS) has manly uniform grain dimensions of 373 ± 110 nm with the lowest degree of agglomerate formation. The opposite was found for samples Mg_2_SiO_4_ (MS) and CaSiO_3_ (W) samples where two groups of particles were noticed: larger particles with grain dimensions of 650 ± 50 nm (dominant) for the Mg_2_SiO_4_ sample (dominant), and 670 ± 100 nm (less noticeable) for the CaSiO_3_ sample; and smaller particles with grain dimensions of 316 ± 20 nm for the Mg_2_SiO_4_ sample, and 270 ± 50 nm for the CaSiO_3_ sample. A more uniform grain size distribution was obtained for the Ca_3_SiO_5_ (TS) sample where larger particles has grain diameters of 416 ± 82 nm, while smaller particles had grain diameters of 226 ± 95 nm. The grain size distributions represent the parameter that affects the particles packaging in epoxy resin and consequently the mechanical properties.

### 3.2. Tensile Properties of the Prepared Composite Materials

The overall comparison of tensile test results (tensile strength, strain, and modulus of elasticity) and toughness of plain epoxy (PE) and 12 different silicate reinforced epoxy resin composite materials are given in Table 4. Seven samples of each material were evaluated, and the minimum and maximum values were discarded for computing the mean values presented in Table 4.

**Figure 7:**
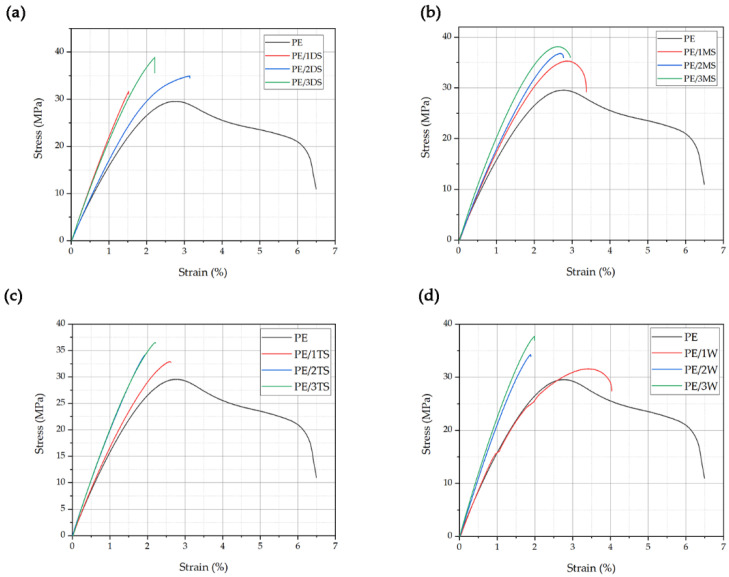
Comparison of stress–strain curves of PE and (**a**) PE/1DS, PE/2DS, PE/3DS; (**b**) PE/MS, PE/2MS, PE/3MS; (**c**) PE/1TS, PE/2TS, PE/3TS; and (**d**) PE/1W, PE/2W, PE/3W specimen.

Figure 7 shows a comparison of the measured tensile stress–strain curves. It was concluded that silicates loading caused improvement of the mechanical properties of the cured composites. Obtained improvements in the mechanical properties of the composites were adjusted to the silicates’ functionalization and morphology, and loading degree. As it can be seen from Table 4, the highest addition of filler loading led to the highest improvement of the tensile strength (23.5% to 31.5% higher values than for the neat epoxy). The sample PE/3DS showed the highest value of tensile strength as it was expected considering the functionality (hydroxyl group amount) and morphology of the silicate filler. DS particles showed the most uniform grain dimensions of 373 ± 110 nm with a lower degree of agglomerate formation (Figure 6), and also the highest amounts of hydroxyl groups suitable for interactions with epoxy polymer chains (Figure 4). Similar was found for the PE/3MS sample, with a slight lowering in tensile strength values due to non-uniform grain size distribution and plenty of larger particles with grain dimensions of 650 ± 50 nm. The samples PE/3TS and PE/3W showed the lowest influence on mechanical properties due to the lowest functionality/hydroxyl groups (Figure 4) and unfavorable grain size distribution (Figure 6). The authors have conducted tensile testing of the specimen reinforced using 5% of fillers. However, the results showed no significant changes in values of tensile strength (less than 1%) compared to the specimen with 3% reinforcement.

Tensile testing enabled obtaining the toughness values of the tested materials. The toughness values (Table 4) of silicate reinforced composites have shown a significant decrease in comparison to plain epoxy.

### 3.3. Digital Image Correlation

The Digital Image Correlation (DIC) approach was used to determine the full-field strain and displacement maps throughout the whole specimen surface. This method is a well-known non-contact optical–numerical approach for evaluating displacement fields [46]. Aramis software was used to determine the von Mises strain of every tested specimen as the local comparable plastic strain because elastic areas are minor in comparison to plastic regions. The following figures obtained using 3D DIC show strain distributions in the *y*-direction that is a direction of applied tensile force. A color scale bar ranging from minimal to maximum values of strain, with photos indicating the instant before fracturing of composites in the experiment depicts the obtained results. The highest values of deformation strains in the *y*-direction are shown by the red spots in the gauge length.

**Figure 8:**
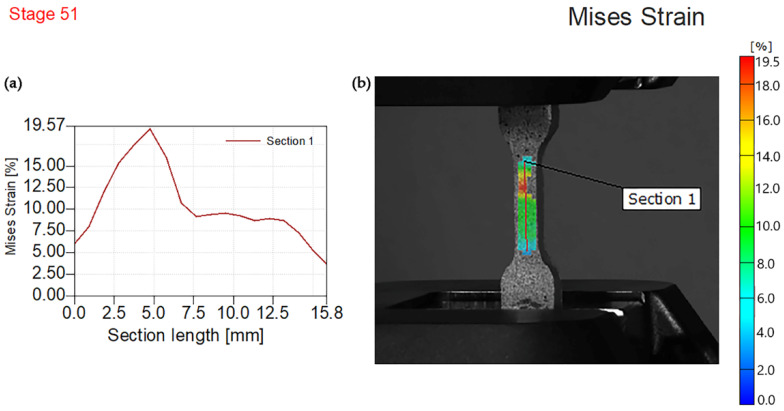
Experimental von Mises strain for the maximum force of 480 N, (**a**) von Mises strain—Section length, (**b**) sample image with the overlaying von Mises strain field.

Figure 8 displays the von Mises deformation results for neat epoxy at a maximum force of 480 N. Section 1 is oriented vertically with a length of 15.8 mm. The highest value of von Mises strain was exhibited at 19.57% and section length at approximately 4.5 mm and as one red point near the middle of the sample surface.

**Figure 9:**
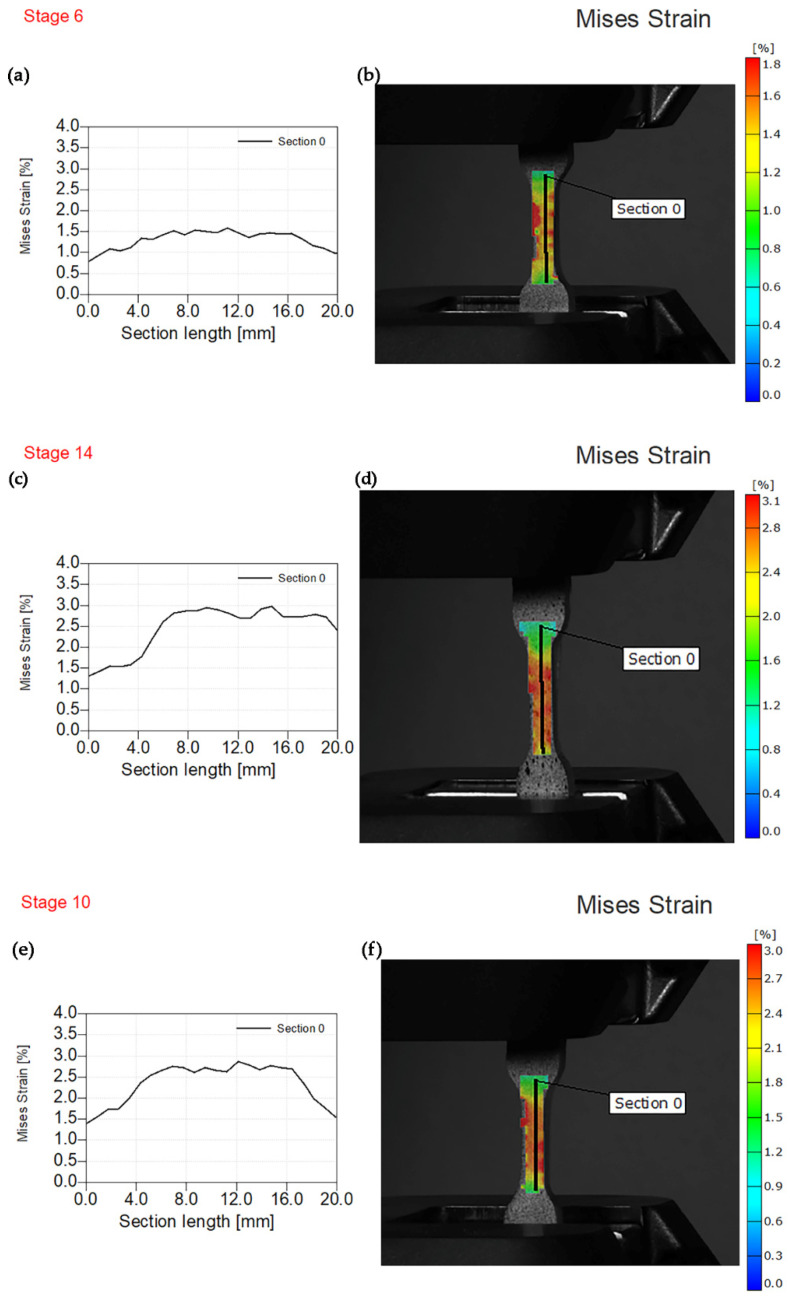
Experimental von Mises strain before fracture. (**a**) Von Mises strain—section length for PE/1DS. (**b**) Sample image PE/1DS with the overlaying von Mises strain field. (**c**) Von Mises strain—section length for PE/2DS. (**d**) Sample image PE/2DS with the overlaying von Mises strain field. (**e**) Von Mises strain—section length for PE/3DS. (**f**) Sample image PE/3DS with the overlaying von Mises strain field.

Figure 9 depicts von Mises strain results for specimen reinforced using dicalcium silicate fillers (PE/1DS, PE/2DS, and PE/3DS). Figure 9a,b show obtained results after testing PE/1DS specimen at a maximum force of 670 N. The highest von Mises strain value was approximately 1.8%, while the section length was 20 mm. The sample image (Figure 9b) shows multiple red spots non-homogenously distributed at the surface area. However, the results obtained after tensile testing of PE/2DS composite materials show different results at the highest value of the tensile force of 730 N (Figure 9c,d). The highest value of von Mises strain was 3.1% and red spots in the gauge length were uniformly distributed due to the highest percent of added fillers. Similar results were obtained at 3% filler content (Figure 9e,f) at a maximum force of 831 N. Higher filler content led to better stress distribution among the particles.

**Figure 10:**
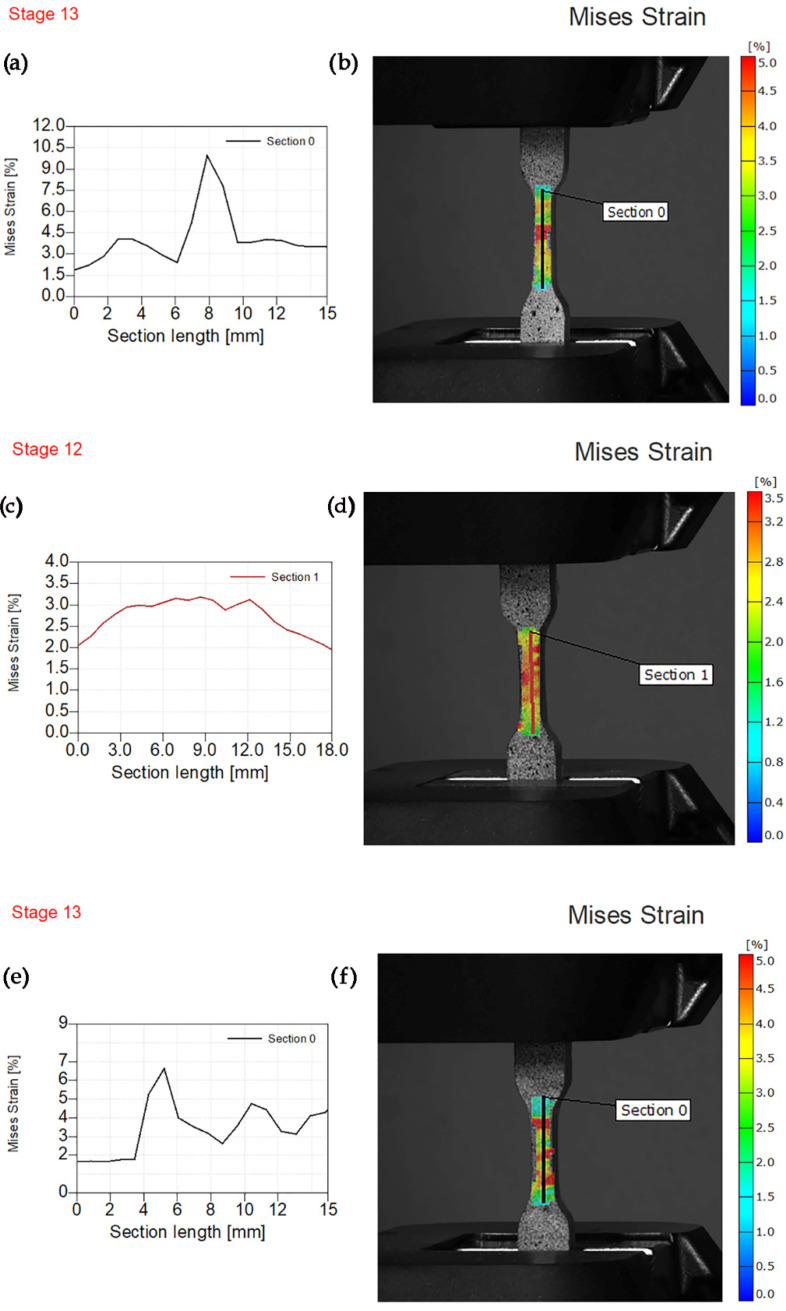
Experimental von Mises strain before fracture. (**a**) Von Mises strain—section length for PE/1MS. (**b**) Sample image PE/1MS with the overlaying von Mises strain field. (**c**) Von Mises strain—section length for PE/2MS. (**d**) Sample image PE/2MS with the overlaying von Mises strain field. (**e**) Von Mises strain—section length for PE/3MS. (**f**) Sample image PE/3MS with the overlaying von Mises strain field.

Von Mises strain measurements for specimens reinforced with magnesium silicate fillers (PE/1MS, PE/2MS, and PE/3MS) are shown in Figure 10. Figure 10 depicts a sharp peak at section length of 8 mm and von Mises strain of 10.4% at a maximum force of 730 N. This was confirmed by the obtained image of the specimen and red area in the middle of the gauge length as a concentrator of applied tensile force. Stress concentration and highest values of strain for PE/2MS and PE/3MS specimen at highest values of the stress of 726, and 770 N, respectively, were also observed.

**Figure 11:**
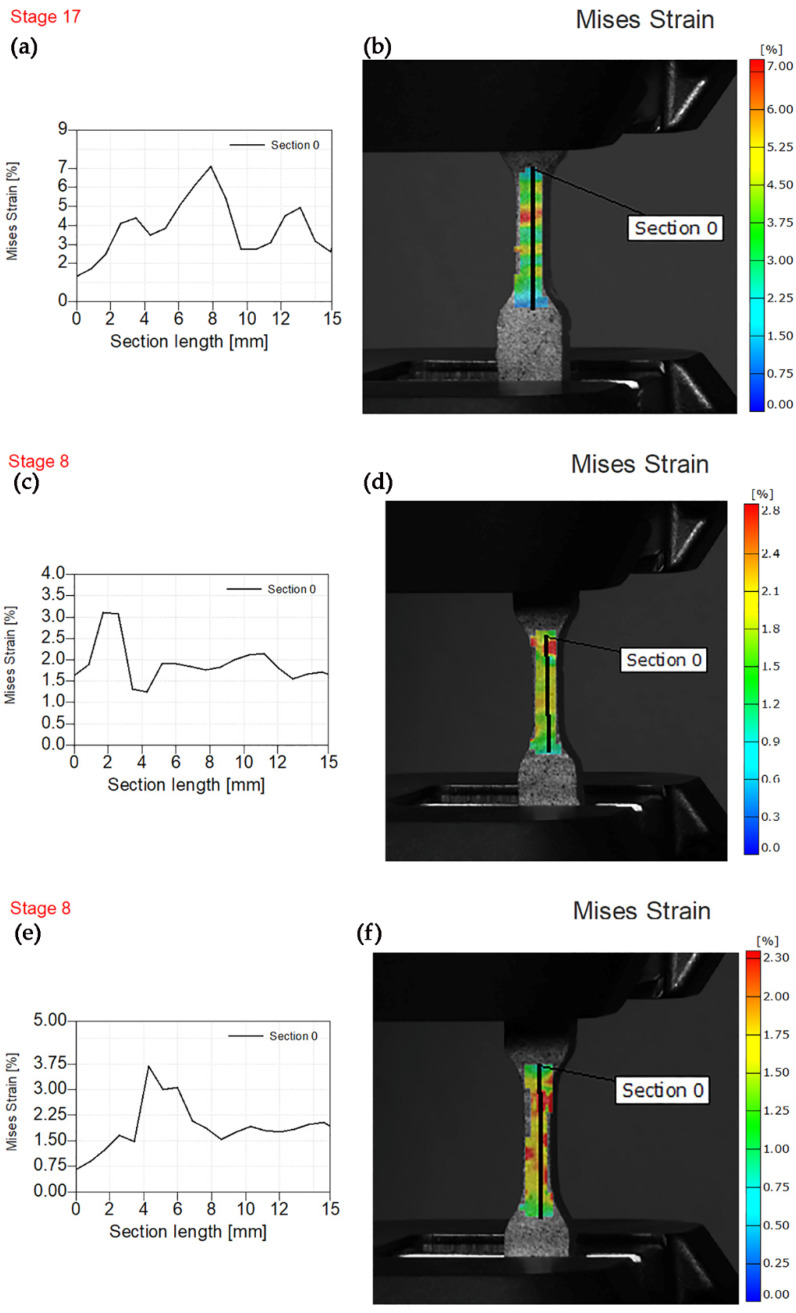
Experimental von Mises strain before fracture. (**a**) Von Mises strain—section length for PE/1TS. (**b**) Sample image PE/1TS with the overlaying von Mises strain field. (**c**) Von Mises strain—section length for PE/2TS. (**d**) Sample image PE/2TS with the overlaying von Mises strain field. (**e**) Von Mises strain—section length for PE/3TS. (**f**) Sample image PE/3TS with the overlaying von Mises strain field.

However, more homogenous stress distribution was observed at 2% of filler content. Two to three specimens exhibited specific behavior during tensile testing, exhibiting three stress concentration areas in the gauge length and highest tensile strength. Similar behavior at 1% filler content was observed during tensile testing of composites reinforced using tricalcium silicate particles (Figure 11a,b). Figure 11c,d show the behavior of composites reinforced using 2% of tricalcium fillers.

**Figure 12:**
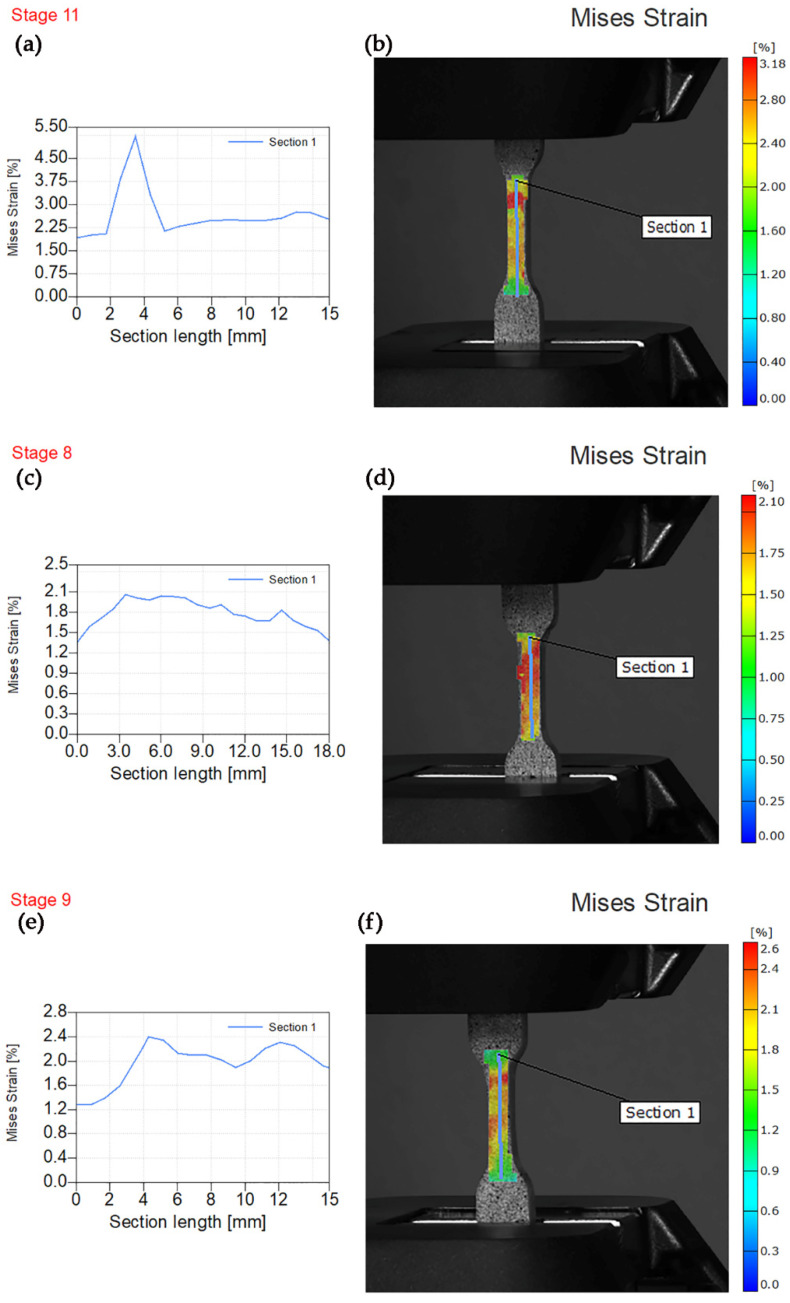
Experimental von Mises strain before fracture. (**a**) Von Mises strain—section length for PE/1W. (**b**) Sample image PE/1W with the overlaying von Mises strain field. (**c**) Von Mises strain—section length for PE/2W. (**d**) Sample image PE/2W with the overlaying von Mises strain field. (**e**) Von Mises strain—section length for PE/3W. (**f**) Sample image PE/3W with the overlaying von Mises strain field.

The highest value of Mises strain of 3.1% was observed at gauge length 1.5–2.5 mm concentrated non-uniformly but in a stress concentration red spot. However, the specimen reinforced using 3% of tricalcium silicate fillers exhibited similar behavior to the specimen reinforced using magnesium silicate fillers at the highest applied force of 800 N. The influence of wollastonite on values of von Mises strain at maximum force is shown in Figure 12.

The lowest value of von Mises strain was observed for PE/2W specimen and uniform stress concentration along the entire gauge length. Nevertheless, 1% of wollastonite fillers lead to the appearance of a sharp peak at 3.5 mm of gauge length with a value of von Mises strain of 5.50%. The maximum value of von Mises strain for PE/3W specimens was 2.4% at a gauge length of 4 and 12 mm.

### 3.4. DMA Analysis

Viscoelastic properties of cured thermosetting polymers are usually investigated using DMA as a standard technique suitable for application in a wide temperature range [47]. Interfacial interactions between fillers and polymer matrices are essential in determining bulk properties and service performances of corresponding composites.

**Figure 13:**
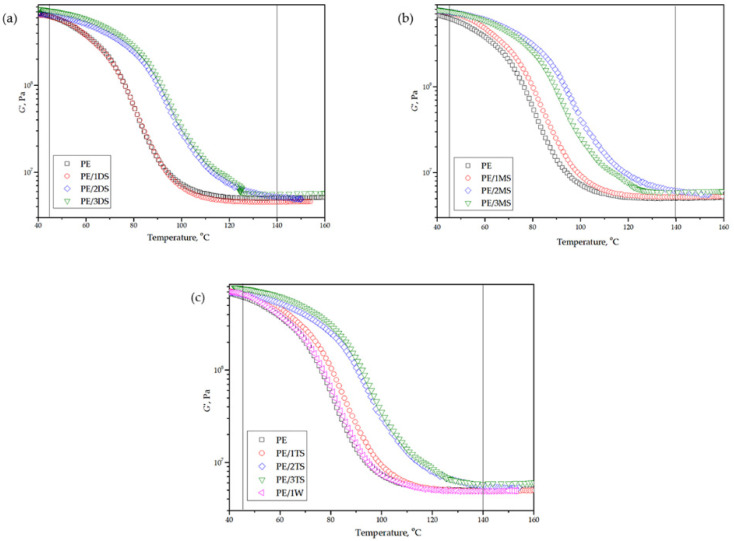
Temperature dependence of storage modulus (G’) of plain epoxy and corresponding composites for (**a**) PE, PE/1DS, PE/2DS, and PE/3DS (**b**) PE, PE/1MS, PE/2MS, PE/3MS (**c**) PE, PE/1TS, PE/2TS, PE/3TS, and PE/1W.

**Figure 14:**
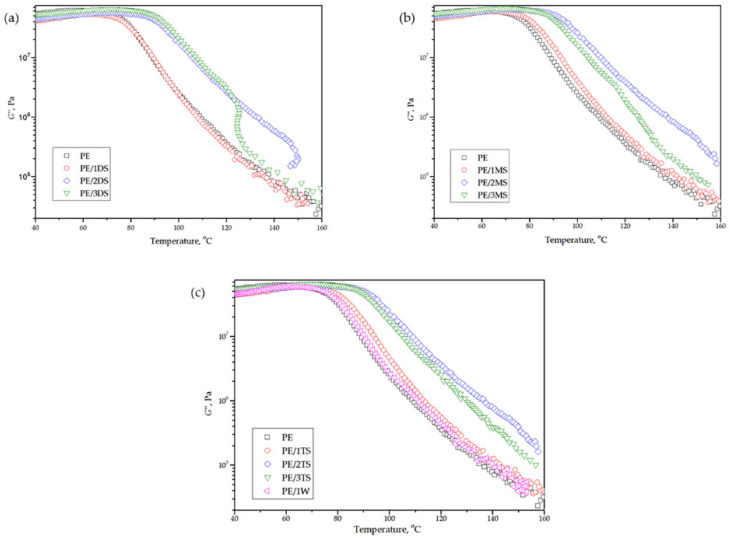
Temperature dependence of loss modulus (G’’) of plain epoxy and corresponding composites for (**a**) PE, PE/1DS, PE/2DS, and PE/3DS (**b**) PE, PE/1MS, PE/2MS, PE/3MS (**c**) PE, PE/1TS, PE/2TS, PE/3TS, and PE/1W.

**Figure 15:**
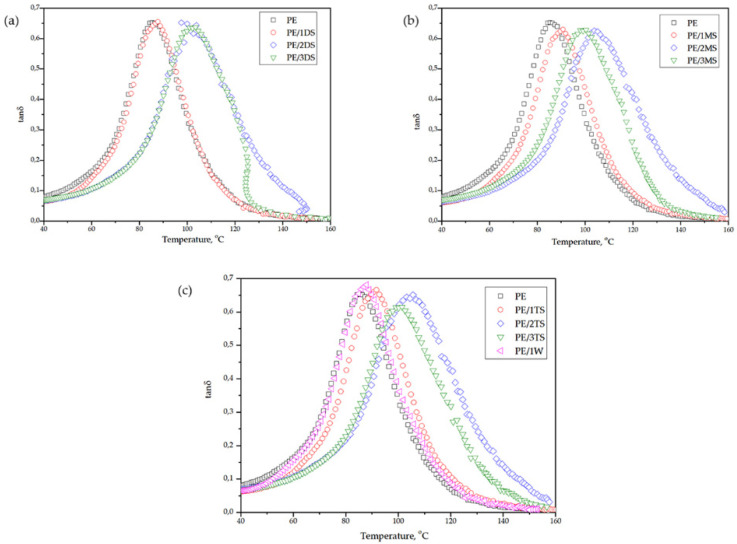
Temperature dependence of *tanδ* of plane epoxy and corresponding composites for (**a**) PE, PE/1DS, PE/2DS, and PE/3DS (**b**) PE, PE/1MS, PE/2MS, PE/3MS (**c**) PE, PE/1TS, PE/2TS, PE/3TS, and PE/1W.

Storage modulus-(G’), loss modulus-(G’’), and damping factor-temperature (*tanδ*) curves of plain epoxy and corresponding composites are shown in Figure 13, Figure 14 and Figure 15. G’ reflects elastic, while G’’ reflects the viscous behavior of epoxy matrix and composites.

Table 5 shows values of G’ in the glassy state and rubbery plateau (G’_GS_ and G’_RP_, respectively), glass transition temperature (*T*_g_), and *tanδ* peak height.

**Figure 16:**
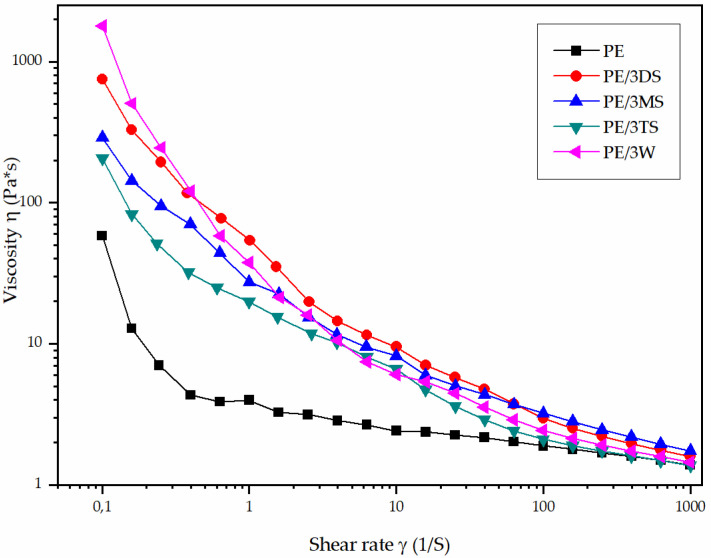
The variation of viscosity as a function of shear rate for a pure epoxy matrix and epoxy suspensions containing 3.0 wt % nanofiller. The experiment was conducted at 25 °C.

The rheology of the uncured epoxy matrix and the nanofillers suspensions is shown in Figure 16 as a plot of viscosity dependence versus shear rate. The data were obtained using a forward shear rate sweep with a measurement time at each shear rate step of 10 s. During the dispersion/homogenization of synthesized nanofillers (silicates) in the liquid epoxy matrix, simultaneous coagulation (or aggregation) and fragmentation by epoxy resin shear are encountered. It can be concluded that viscosity of the all investigated samples decreases with increasing the shear rate, which may be caused by shear-induced heating. The flow behavior of suspensions with 3 wt % nanofiller loading showed deviation compared to the pure epoxy matrix due to different morphology and a hydroxyl functionality of the nanofiller. Aggregates formed by MS and W nanofillers immobilize polymer chains of the epoxy matrix and therefore increased the effective volume of the nanofiller particles in the epoxy suspension, and due to that, the increase of suspension viscosity is a consequence. Moreover, the plenty of hydroxyl functionalities in MS and DS nanoparticle surfaces contribute to the somewhat increase of the suspension viscosity. The hydrogen bonding interaction plays an important role in viscosity change at a higher shear rate for epoxy suspension with 3.0 wt % of DS nanofiller, even those particles with the most uniform size distribution. Such behavior is opposite to the viscosity change of PE/3W showing the highest viscosity decrease: a significant effect of agglomeration degree at low shear rate is negligible at higher shear rate. EP/3TS suspension showed the lowest viscosity change compared to the pure epoxy resin due to low functionality and low rate of the hydrogen bonding interaction with epoxy matrix and particle size in the range from 416 ± 82 nm to 226 ± 95 nm.

### 3.5. TEM Analysis

**Figure 17:**
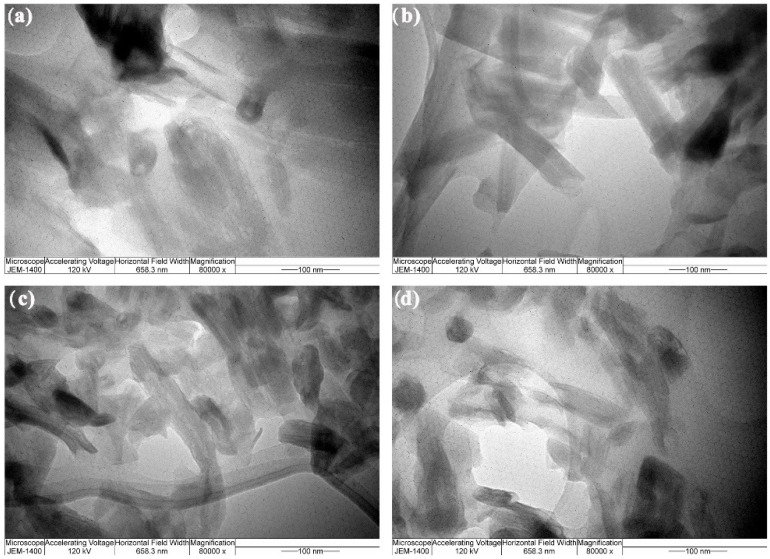
TEM images of (**a**) Ca_2_SiO_4_; (**b**) Mg_2_SiO_4_; (**c**) Ca_3_SiO_5_; and (**d**) CaSiO_3_.

TEM images of composites with a filler content of 3% revealed a larger number of needle-like formations that were nearly identical in size and form and were grouped in an uneven pattern (Figure 17).

## 4. Discussion

The chemical and crystalline structure of the prepared silicate fillers were validated by the FTIR and XRD results. FTIR spectra of Mg_2_SiO_4_ and Ca_2_SiO_4_ confirmed the presence of hydroxyl groups’ vibrational characteristics by bands at 1600 to 1450 cm^−1^ and 3641 cm^−1^. The presence of hydroxyl groups affects the degree of functionality of the particles/fillers, which is responsible for the creation of hydrogen bonding interactions with the cured epoxy matrix and contributes to nanocomposite reinforcing. The samples’ average crystallite size was estimated to be around 20 nm by the Rietveld refinement approach. SEM images of the prepared silicates confirmed a needle-like structure and nanometer and submicron dimensions. The nanoparticle packaging in epoxy resin and, as a result, the mechanical characteristics are affected by these grain size distributions.

Tensile strength has increased with the addition of fillers to the epoxy matrix, according to tensile test results. Based on the obtained results, tensile strength has increased for all the manufactured composites. After the addition of 1%, then 2% and finally 3% of particles, the tensile strength of the composites rose gradually. Composites with wollastonite filler loading (PE/1W, PE/2W, PE/3W) showed the lowest tensile strength increasement up to 23.5%, followed by tricalcium silicate reinforced composites (PE/1TS, PE/2TS, PE/3TS) with 27.5% tensile strength increasement. Magnesium silicate (PE/1MS, PE/2MS, PE/3MS) and dicalcium silicate (PE/1DS, PE/2DS, PE/3DS) composites showed higher values of tensile strength increased by 29.0% and 31.5%, respectively. The silicates’ functionalization and morphology, as well as the loading degree, were used to enhance the mechanical properties of the prepared composites. Following Figure 6, it was shown that dicalcium silicate particles had the most uniform particle diameters and the least degree of agglomeration, as well as the maximum number of hydroxyl groups suited for interactions with epoxy polymer chains that lead to better tensile properties of the obtained composite in comparison to plain epoxy. Similar results were reported for the PE/3MS sample, with a minor decrease in tensile strength due to non-uniform grain size distribution and a substantial number of bigger particles with grain dimensions twice the dimensions of dicalcium silicates’ dimensions. Due to the use of the lowest functionality/hydroxyl groups (Figure 4) and an unfavorable particle size distribution of tricalcium silicates and wollastonite, samples PE/3TS and PE/3W had the least impact on mechanical characteristics shown in Figure 6.

Crack arrest mechanisms working at nanometer or sub-nanometer scales of nanofiller, such as partly agglomerated nanoparticles resulting in localized stiffness gradients, might have caused a significant decrease of toughness values was observed for all silicate rein-forced composites in comparison to plain epoxy. Therefore, it could be stated that the appropriate contribution of a couple of factors: nanofiller agglomeration and size, as well as surface functionalities, determine the trend of the toughness change of studied nanocomposites [48].

The strain distributions in the *y*-direction, which is the direction of applied tensile force, were analyzed using the 3D DIC method. The findings for all specimens examined led to the conclusion that the low level of fillers in the material caused the emergence of unique stress concentrators near or in the center of the gauge length. Higher filler content, on the other hand, resulted in improved stress distribution among particles and along the gauge length. However, the performance of added fillers is directly dependent on the interaction of fillers and epoxy matrix.

The TEM image presented in Figure 17a displays particles of Ca_2_SiO_4_ uniformly dispersed and resembling the homogenous dispersion of Mg_2_SiO_4_ shown in Figure 17b. Non-homogenous dispersion was observed for Ca_3_SiO_5_ (Figure 17c) and CaSiO_3_ (Figure 17d). The hydroxyl (OH) groups found in epoxy resin are thought to have essential chemical interactions with a role to increase binding strength. It appears that the pending hydroxyl groups in the structure of Mg_2_SiO_4_ and Ca_2_SiO_4_ became involved in the polymer matrix in the development of intramolecular hydrogen bonding, which influenced the strength of the connection between the constituents in composites and reinforced the polymer matrix. The OH groups present in the filler structure (Figure 4) appear to have catalyzed polymerization and facilitated epoxy and curing agent molecule diffusion between the nanofillers [49,50]. In the end, these interactions led to different filler–matrix interactions and composite with enhanced reinforcement proven by the obtained tensile testing results (Table 4).

From the G’-temperature and G’’-temperature curves (Figure 13 and Figure 14), it is evident that values of G’ are higher compared to the values of G’’ in the whole temperature region. Additionally, composites display higher values of the G’ in a glassy state compared to the plane epoxy. Such a phenomenon indicates that the incorporation of fillers has slightly improved the stiffness of the corresponding composites. The same increasing trend of G’ values is observed in a rubbery plateau. A decrease in values G’ of all samples is associated with an increase in temperature which is caused by intensive movement of the epoxy chains. Composites with 2.0 and 3.0 wt % loading of the fillers display a smaller decrease in G’ values with temperature increases, which suggests that those composites possess a more homogenous structure. Composites reinforced using wollastonite fillers showed similar results as composites reinforced using tricalcium silicate fillers.

The G’’-temperature curve shows a slight rise of G’’ values below the transition region after what values significantly decrease with temperature increase, which is attributed to internal friction which promotes energy dissipation caused by the presence of reinforcements [47]. The *tanδ* height values are similar for plane epoxy and corresponding composites and amounts from 0.62 to 0.68. The glass transition temperature (*T*_g_) is determined from the *tanδ* peak position (Figure 15) and it reaches a value of 85.4 °C for plane epoxy, and increases from 87.8 to 105.6 °C for corresponding composites. The increase in *T*_g_ for composites occurs due to the immobilization of the epoxy macromolecular chains near the surface of the fillers and strong interfacial interaction between the epoxy chains and all types of the used fillers.

The viscosity of all the samples tested reduces as the shear rate increases, which might be due to shear-induced heating. The flow behavior of suspensions containing 3 wt % nanofillers differed from that of the pure epoxy matrix due to the nanofillers’ distinct morphological properties and hydroxyl functionality. The presence of aggregated magnesium silicate and wollastonite nanofillers leads to an increase in suspension viscosity. Furthermore, the larger rise in suspension viscosity was influenced by the presence of hydroxyl groups at the surface of magnesium silicate nanofillers. The viscosity change at low shear rates of epoxy solution with 3 wt % dicalcium silicate nanofillers (DS) was influenced by the appearance of H-bonding interactions playing the most critical impact. Finally, as compared to pure epoxy resin, the epoxy solution with 3 wt % percent tricalcium silicate nanofillers (TS) had the lowest viscosity change due to low functionality and a low rate of H-bond interaction with epoxy matrix, as well as particle size in the range from 416 ± 82 nm to 226 ± 95 nm.

## 5. Conclusions

The purpose of this work was to analyze tensile properties of composite materials reinforced using four different fillers: dicalcium silicate, magnesium silicate, tricalcium silicate, and wollastonite. Full-field non-contact 3D DIC was proposed to mitigate the constraints of traditional experimental methods, and to detect full-field displacement and strain. The tensile strength of the composites increased steadily after the addition of 1%, 2%, and finally 3% of fillers. In respect to plain epoxy, the addition of 3% dicalcium silicate, magnesium silicate, tricalcium silicate, and wollastonite increased tensile strength by 31.51%, 29.01%, 27.49%, and 23.47%, respectively. The low level of fillers in the material induced the appearance of distinct stress concentrators near or in the center of the gauge length, according to the findings for all specimens evaluated. The relationship between fillers with epoxy matrix influenced the efficiency of additional nanofillers: hydroxy groups present in the chemical structure of Mg_2_SiO_4_ and Ca_2_SiO_4_ enhanced the distribution of fillers and stronger bonding between these nanofillers and epoxy matrix, which was proven by the TEM images. Tensile properties of the obtained composites were also influenced by the grain size distribution and agglomeration. The highest enhancement of tensile properties was observed for the calcium disilicate reinforced epoxy composites due to their lowest particle dimensions and the lowest degree of agglomeration. However, the lowest enhancement of tensile properties was observed for the materials reinforced using wollastonite nanofillers because of poor functionality (lack of hydroxyl groups) and an undesirable grain size distribution.

The presence of intramolecular interactions between fillers and polymer matrix was confirmed by DMA analysis: the increase in *T*_g_ for composites was caused by the immobilization of epoxy macromolecular chains near the surface of the fillers and a strong intermolecular interaction between the epoxy chains and all types of filler utilized.

The effects of nanofillers on the viscosity of a liquid epoxy matrix were studied. Although nanofillers have a substantial influence on epoxy resin viscosity, the difference between their effects is minimal which was further discussed in the manuscript. It was concluded that the functionality of the nanofillers and the formation of agglomerates in the epoxy matrix affected the viscosity of the prepared materials.

## Figures and Tables

**Table 1:** The main properties of epoxy resin and hardener [34,35,36].

Component	Properties
Araldite GY 250	Unmodified Liquid Epoxy Resin Epoxy value: 5.30–5.55 Eq/kg Weight per epoxide: 180–189 g/Eq Viscosity at 25 °C: 10,000–12,000 mPa s Density at 25 °C: 1.17 g/cm^3^ Flash point: ≥200 °C
Araldite DY-E	Monofunctional, aliphatic, reactive diluent for epoxy resins Epoxy index: 3.15–3.60 Eq/kg Epoxy equivalent: 278–317 g/Eq Viscosity at 25 °C: 4–12 mPa s Density at 20 °C: 0.89 g/cm^3^ Flash point: ~150 °C
Aradur 2963-1	Modified cycloaliphatic polyamine Amine number: 325–350 mg KOH/g Viscosity at 25 °C: 30–70 mPa s Density at 25 °C: 1.00 g/cm^3^ Flash point: ≥108 °C

**Table 2:** Sample symbols, nanofillers and nanofiller content.

Sample Symbol	Nanofiller	Nanofiller Content, %
PE	-	-
PE/1DS	Ca_2_SiO_4_	1
PR/2DS	Ca_2_SiO_4_	2
PE/3DS	Ca_2_SiO_4_	3
PE/1MS	Mg_2_SiO_4_	1
PE/2MS	Mg_2_SiO_4_	2
PE/3MS	Mg_2_SiO_4_	3
PE/1TS	Ca_3_SiO_5_	1
PE/2TS	Ca_3_SiO_5_	2
PE/3TS	Ca_3_SiO_5_	3
PE/1W	CaSiO_3_	1
PE/2W	CaSiO_3_	2
PE/3W	CaSiO_3_	3

**Table 3:** Mean crystallite size and structural parameters of the obtained silicate fillers.

	Mg_2_SiO_4_	CaSiO_3_	Ca_2_SiO_4_	Ca_3_SiO_5_
ICDD	01-085-1357	01-084-0655	01-076-3608	00-031-0301
Crystal structure	Orthorhombic	Monoclinic	Monoclinic	Anorthic
Space group	Pmnb (62)	P21/a (14)	P21/n (14)	P1 (1)
Crystallite size, nm	17.2	28.38	13.54	5.38
Strain	0.30	0.08	0.23	0.44
Rwp	12.63	9.28	9.28	6.19
Rp	6.92	6.37	5.92	4.46
Re	3.31	3.82	3.87	3.89
GOF	3.8215	2.4313	2.399	1.5895
a (Å)	4.7523	15.4529	5.512	14.08
b (Å)	10.213	7.3416	6.781	14.423
c (Å)	5.9819	7.0732	9.309	24.822

**Table 4:** Tensile properties of plain epoxy and silicate reinforced composites.

Sample	Max. Stress MPa	Max. Strain %	Modulus of Elasticity GPa	Toughness kJ/m^3^
PE	29.6 ± 0.6	6.85 ± 0.15	1.50 ± 0.03	1165 ± 24
PE/1DS	31.7 ± 0.7	1.53 ± 0.03	2.21 ± 0.05	206 ± 5
PE/2DS	34.9 ± 0.7	3.15 ± 0.07	1.6 ± 0.03	573 ± 11
PE/3DS	38.9 ± 0.7	2.23 ± 0.04	2.12 ± 0.04	398 ± 7
PE/1MS	35.3 ± 1.0	3.39 ± 0.09	1.70 ± 0.05	648 ± 18
PE/2MS	36.8 ± 0.9	2.77 ± 0.07	1.8 ± 0.04	499 ± 12
PE/3MS	38.2 ± 0.9	2.96 ± 0.07	2.02 ± 0.05	600 ± 14
PE/1TS	32.9 ± 0.7	2.80 ± 0.06	1.6 ± 0.03	423 ± 9
PE/2TS	34.1 ± 0.6	2.06 ± 0.04	1.96 ± 0.03	294 ± 5
PE/3TS	36.5 ± 0.9	2.21 ± 0.06	2.04 ± 0.05	374 ± 9
PE/1W	31.6 ± 0.6	4.04 ± 0.08	1.44 ± 0.03	738 ± 14
PE/2W	34.3 ± 0.7	1.89 ± 0.04	2.14 ± 0.04	295 ± 6
PE/3W	37.7 ± 1	1.99 ± 0.05	2.25 ± 0.06	346 ± 9

**Table 5:** DMA results of plain epoxy and corresponding composites.

Sample	*G*’_GS45°C_, MPa	*G*’_RP140C_, MPa	*T*_g_ (*tanδ* Peak, °C)	*tanδ* Peak Height
**PE**	627.99	5.01	85.4	0.65
**PE/1DS**	613.52	4.54	87.8	0.65
**PE/2DS**	640.49	5.22	103.8	0.63
**PE/3DS**	725.58	5.62	102.2	0.64
**PE/1MS**	688.42	4.95	90.8	0.63
**PE/2MS**	690.00	5.73	103.9	0.63
**PE/3MS**	750.25	5.91	99.1	0.62
**PE/1TS**	664.28	4.88	99.7	0.67
**PE/2TS**	686.22	5.48	105.6	0.65
**PE/3TS**	767.14	5.88	100.2	0.61
**PE/2W**	658.82	4.87	87.9	0.68

## Data Availability

The data presented in this study are available on request from the corresponding author.
